# The *Plasmodium* Export Element Revisited

**DOI:** 10.1371/journal.pone.0001560

**Published:** 2008-02-06

**Authors:** Jan Alexander Hiss, Jude Marek Przyborski, Florian Schwarte, Klaus Lingelbach, Gisbert Schneider

**Affiliations:** 1 Johann Wolfgang Goethe-University, Institute of Cell Biology and Neuroscience, Centre for Membrane Proteomics, Frankfurt am Main, Germany; 2 Faculty of Biology, Philipps-University Marburg, Marburg, Germany; Uppsala University, Sweden

## Abstract

We performed a bioinformatical analysis of protein export elements (PEXEL) in the putative proteome of the malaria parasite *Plasmodium falciparum.* A protein family-specific conservation of physicochemical residue profiles was found for PEXEL-flanking sequence regions. We demonstrate that the family members can be clustered based on the flanking regions only and display characteristic hydrophobicity patterns. This raises the possibility that the flanking regions may contain additional information for a family-specific role of PEXEL. We further show that signal peptide cleavage results in a positional alignment of PEXEL from both proteins with, and without, a signal peptide.

## Introduction

*Plasmodium falciparum* (*P.falciparum*) is an intracellular parasite of the human red blood cell and the cause of the most virulent form of malaria. The severe pathology is at least partly a result of a modification of the host cell plasma membrane by proteins synthesized and exported by the parasite [1]. From a cell biological point of view this is unusual, as the non-infected human erythrocyte lacks a machinery to facilitate directed protein transport. An additional obstacle is the location of the parasite within a so-called “parasitophorous vacuole” (PV), which separates the parasite from the host cell cytosol, the vacuolar membrane thereby forming a further barrier for proteins destined for the host cell. In two elegant studies, Hiller *et al.* [2] and Marti *et al.* [3] identified a short peptide sequence, referred to as the vacuolar transport signal (VTS) or *Plasmodium* export element (PEXEL), respectively. This motif is frequently found in parasite proteins that are transported beyond the confines of the vacuolar membrane. Although VTS and PEXEL differ slightly in their structure, they share the conserved five-residue motif Rx(L,I)x(D,E,Q).

Dominant protein families of the *P.falciparum* “exportome” are parasite-encoded surface proteins such as the erythrocyte membrane protein 1 (PfEMP1)–the major *P.falciparum* virulence factor [4]–[6]–and the RIFIN and STEVOR surface antigen families [7], [8]. We found that 28% of the putative *P. falciparum* proteome contain the PEXEL/VTS pattern. This is a large number of proteins, and raises the question whether the presence of the motif is the sole defining criterion for exported parasite proteins. In fact, residues surrounding the PEXEL motif were found to be important in correct trafficking or folding of exported proteins [9], [10], and a recent study suggests that the short pentameric core motif alone is insufficient to cause protein traffic across the PV membrane [11]. Apparently, additional factors need to be taken into account when predicting the size and members of the *Plasmodium* exportome as well as antigens at the surface of the infected erythrocyte. This hypothesis is substantiated by the observation that members of the RIFIN protein family locate in different cellular compartments despite the fact that all members of the RIFIN protein family contain a PEXEL sequence: A-type RIFINs are transported to the surface of infected erythrocytes *via* Maurer's clefts, whereas B-type RIFINs remain inside the parasite [12]. Wahlgren and coworkers already speculated that residue positions in the PEXEL motif and additional family-specific conserved stretches of amino acids are required for differential protein targeting [12], which is in agreement with the studies by Przyborski *et al.* [10]. One question arising from these preliminary findings is whether the PEXEL-flanking sequence regions contain family-specific information.

## Results and Discussion

So motivated, we analyzed residue positions surrounding the PEXEL motif. We compiled a set of 5,571 unique proteins from *P.falciparum*, extracted from PlasmoDB [13], TIGR/NCBI clone 3D7 [14], and EMBL-EBI [15]. Pattern matching with SEEDTOP [16] retrieved 1,557 (28%) sequences containing the PEXEL motif. 412 (7.4%) hits were found by a generalized Hidden-Markov-Model (false-positive rate: 5%), which requires, in addition to the PEXEL motif, a preceding hydrophobic region for prediction of exported proteins [17]. For further analysis, we extracted stretches of 25 amino acids from these 412 predicted proteins containing the central five-residue PEXEL motif and ten additional residues on both sides (data available as supplementary material). When multiple PEXEL motifs existed in one protein sequence, only the most N-terminal occurrence was extracted.

We performed all-against-all pair-wise alignment of the 25-residue fragments using BLAST ([16]; Gapped BLAST was run with the BLOSUM62 matrix, [18], and gap-open cost  = 11, gap-elongation = 1). Only 6% of the sequences aligned to proteins outside their family, and 78% of all fragments aligned to sequences of the corresponding protein family with average *E*-values up to 0.1. These results indicate that the residues flanking the PEXEL motif contain family-specific information. It is evident that the shortness of the sequences used (25 residues), and the failure to align 22% of the sequence fragments limit this approach for general prediction of potentially exported proteins and protein family assignment. It has been argued before that straightforward sequence alignment may not be appropriate to find all members of the *P.falciparum* exportome because individual protein families are particularly deviating in their primary sequences, for example beta-barrel proteins from outer bacterial and organelle membranes [9].

**Figure 1:**
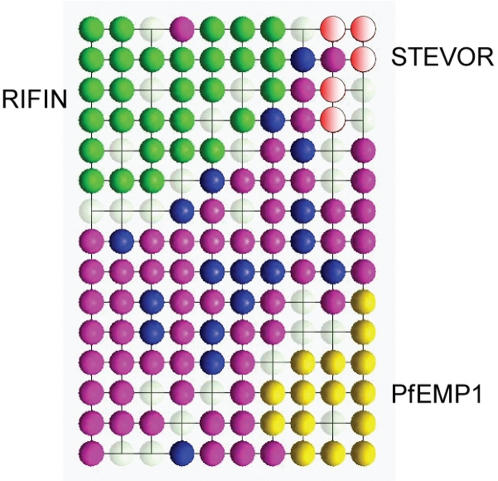
SOM projection of the PEXEL containing sequence fragments from *P.falciparum* proteins. The SOM contains 10×15 topologically ordered data clusters (“neurons”). Locations of RIFIN (green), STEVOR (red), and PfEMP1 (yellow) proteins are highlighted. The location of hypothetical proteins is shown in magenta. Blue color indicates “other” PEXEL-containing fragments. A neuron is assigned one particular class if more than 50% of its clustered proteins belong to one family. White neurons do not contain any proteins (“empty sequence space”). Map generated with the software SOMMER [27].

In a complementary approach, we encoded the sequence fragments by seven physicochemical amino acid properties [19]: hydrophilicity [20] and hydrophobicity [21] scales, volume [22], surface [23], bulkiness, refractivity, and polarity [24]. This led to a 25×7 = 175-dimensional vectorial sequence representation. We employed Kohonen's self organizing map (SOM) technique [25] for visualizing the data distribution by nonlinear projection of this high-dimensional sequence space [26]. As a result of SOM training, the topology of the data distribution is shown on a two-dimensional map, and cluster formation of RIFIN, STEVOR, and PfEMP1 sequences is observed (Figure 1). The physicochemical sequence representation led to a reasonable grouping of the three dominant PEXEL-containing protein families.

Noteworthy, based on relative amino acid frequency only (calculated from full-length sequences), the three protein families cannot be distinguished (Kolmogorov-Smirnov test significance at the 5% level).

The SOM is grounded on a non-deterministic process. Thus the projections slightly differ in repeated runs. We selected a SOM projection with a small mean quantization error. Clustering strength was evaluated by calculating the topological *intra*-family distance of proteins on the SOM. We found that the PEXEL containing families RIFIN (average distance: 4.17), PfEMP1 (average distance: 2.99), and STEVOR (average distance: 2.20) have smaller pair-wise distances than the remaining “hypothetical” proteins (average distance: 5.99). This supports our notion that RIFIN, PfEMP1, and STEVOR form local distributions.

The RIFIN cluster contains two major sub-families, A-RIFIN and B-RIFIN proteins [28]. The average topological distance of SOM neurons containing B-RIFIN is 2.5, and for A-RIFIN 3.5, indicating that B-RIFIN proteins are more similar to each other than the A-RIFINs, based on the sequence fragments analyzed.

The SOM projection was then used to predict the family membership of the remaining 180 PEXEL-containing hypothetical *P.falciparum* proteins. A conservative prediction was performed as we focused only on neurons containing at least 50% members from one protein family. Among the candidate proteins, one co-localizes with the RIFIN, five with the STEVOR, and one with the PfEMP1 family on the SOM (see supplementary material). Noteworthy, these suggested assignments are based on the similarity of the PEXEL motif flanking regions only.

The hypothetical sequences that do not co-cluster with known members of the RIFIN, PfEMP1, and STEVOR families are not necessarily false-positives. They might belong to other PEXEL containing protein families. In our study, we focused only on the three dominant PEXEL-containing protein families from *P. falciparum*. For determination whether they represent actual false-positives with regard to intracellular localization, biological experiments are required. This is beyond the scope of the present study.

The formation of clusters of protein families on the SOM corroborates the hypothesis that family-related information exists in the flanking areas of the PEXEL motif. This would not be without precedent, as precursor proteins targeted to cellular compartments such as the mitochondria and chloroplasts often contain essential protein targeting information at their N-terminus, sometimes encoded on an extra 5′ exon. A similar situation can be found in proteins targeted to the apicoplast of *P. falciparum*, and, *e.g*., in exported *P. falciparum* homologues of the HSP40 chaperone family [29].

The apparent positional conservation of the PEXEL motif (approximately 20 amino acids C-terminal to the hydrophobic sequence, and situated 15-20 amino acids N-terminal to the beginning of the mature protein) has been suggested to be required for correct recognition by the transport machinery [30]. As of today, there is no experimental evidence to suggest that the PEXEL containing region is actually cleaved. N-terminal protein sequencing of exported proteins has been attempted, but so far without success [31]. Additionally, Western blot analysis shows no size difference between proteins within the parasite's secretory pathway and those that have reached the erythrocyte cytosol, although this size shift should be able to be detected [32], [33].

In the present study, we show an apparent family-specific conservation of physicochemical residue profiles for short PEXEL-flanking regions (*vide infra*). This raises the possibility that this region may be more than just a “simple transport signal”, *e.g.* playing a role in alternative transport mechanisms, or in regulation of protein transport. To this end, it is noteworthy that a PEXEL containing RESA-GFP chimera was only correctly transported to its correct sub-cellular location when expressed under control of its endogenous promoter. Expression of the same protein under control of a heterologous promotor led to retention of the reporter within the lumen of the PV [34]. We speculate that the PEXEL-flanking regions might therefore influence regulated secretion of proteins, either temporally, or even in response to external stimuli. In other systems, evidence is also accumulating to suggest that targeting signals, such as endoplasmic reticulum (ER) signals, far from being “just greasy peptides”, can contain important regulatory information [35], [36].

**Figure 2:**
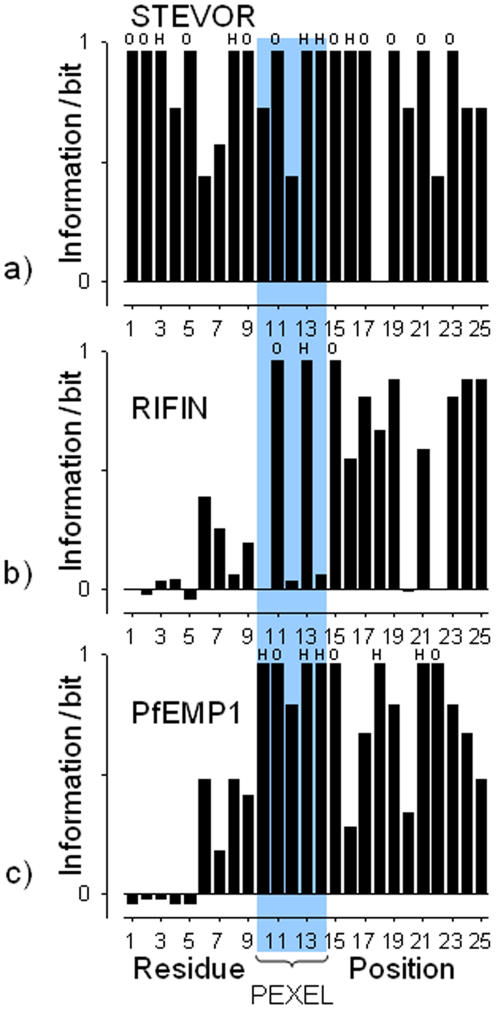
Occurrence of hydrophobic and hydrophilic residues up and downstream the PEXEL motif. Information plot of the PEXEL motif and surrounding residue positions in the protein families STEVOR (*N* = 30) (a), RIFIN (*N* = 125) (b) and PfEMP1 (*N* = 58) (c). Large values indicate sequence positions with conserved hydrophobic (H) or hydrophilic (O) residues (see text for residue classification). The position of the PEXEL motif is highlighted.

In all three *Plasmodium* protein families studied, the downstream flanking regions show high information content with regard to hydrophobic and hydrophilic residues. Noteworthy, the upstream flanking region of the STEVOR examples exhibits additional conserved patterns not present in the known PfEMP1 and RIFIN proteins (Figure 2).

For calculation of the position-specific information content the software H-BloX was used [37] (Eq. 1). The 25-residue fragments were encoded by a two-letter alphabet containing “hydrophobic” (A,C,F,G,I,L,M,T,V,W) and “hydrophilic” residues (D,E,H,K,N,P,Q,R,S,Y).
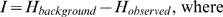
(Eq.1)

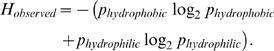



The expected distribution *H_background_* of hydrophobic and hydrophilic residues was calculated from the amino acid distribution found in the predicted *P.falciparum* proteome (in percent: A = 1.9, C = 1.8, D = 6.5, E = 7.2, F = 4.4, G = 2.8, H = 2.4, I = 9.2, K = 11.7, L = 7.5, M = 2.2, N = 14.5, P = 2.0, Q = 2.7, R = 2.6, S = 6.4, T = 4.1, V = 3.9, W = 0.5, Y = 5.7).

Site-directed mutagenesis of charged residues within this region has previously been shown to cause an accumulation of chimeric reporter proteins within the parasite's endoplasmic reticulum [10]. This region is predicted to contain several putative chaperone binding sites, suggesting that disruption of chaperone binding sites may interfere with chaperone mediated protein folding and quality control, leading to an aggregation of incorrectly folded protein, and a corresponding reduction in protein export. Mutation of residues “downstream” of the PEXEL motif had minimal or no effect on the localization of a STEVOR protein [10], highlighting the relative importance of its PEXEL preceding sequence.

We then computed averaged hydrophobicity profiles of PEXEL plus flanking residues for each of the three protein families, using the hydrophilicity scale according to Hopp and Woods [20]. Table 1 gives the correlation coefficients for matching the family-specific profiles against the fragments from the three families. We observe that there is only low cross-family correspondence of the property patterns, again suggesting family specificity of the flanking regions.

**Table 1:** Pearson correlation between family-derived hydrophilicity profiles and 25-residue sequence fragments containing the central PEXEL motif.

*Profile from*	*Fragments from*		
	PfEMP1	RIFIN	STEVOR
PfEMP1	0.75 (0.09)	0.15 (0.09)	0.07 (0.11)
RIFIN	0.15 (0.07)	0.73 (0.08)	0.46 (0.10)
STEVOR	0.07 (0.12)	0.43 (0.09)	0.80 (0.16)

Standard deviation in brackets.

**Figure 3:**
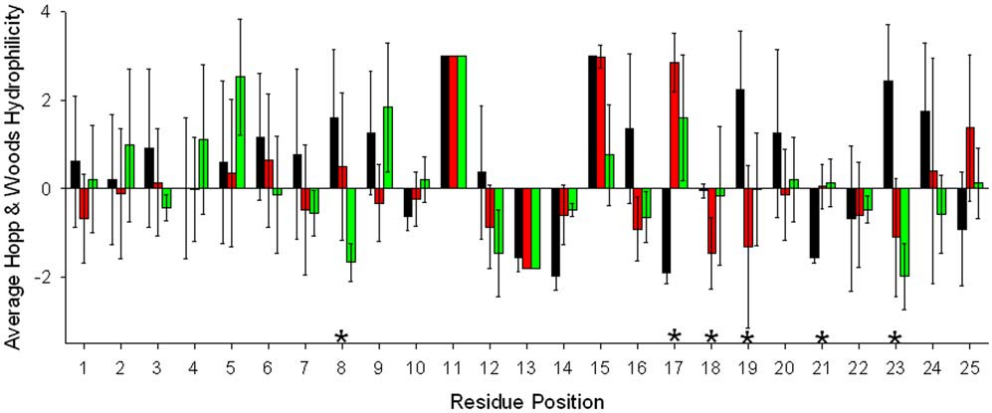
Average position-specific hydrophobicity in 25-residue fragments containing the central PEXEL motif (at positions 11-15). Color indicates the protein family (black: PfEMP1, gray: RIFIN, light gray: STEVOR). Error bars give standard deviations. Asterisks indicate positions characteristic for one of the families. Note that the Hopp & Woods scale [20] is a “hydrophilicity” scale with negative values for hydrophobic residues.

More detailed analysis of the position-specific preference of hydrophobic or hydrophilic residues indicate that position 8 is important for discrimination of STEVOR proteins, whereas positions 17–19, 21, 23 are characteristic of PfEMP1 proteins (Figure 3). Position 18 is dominated by glycine in PfEMP, resulting in high information content (Figure 2) yet a hydrophobicity value of close to zero (Figure 3).

**Figure 4:**
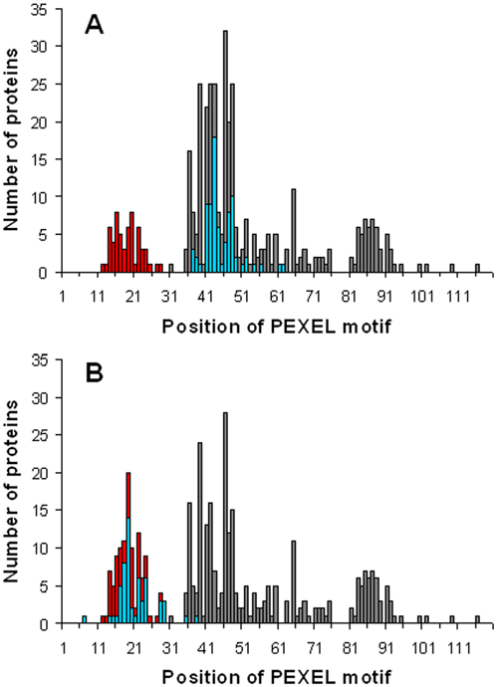
Distribution of the first position of the PEXEL motif. A) position of the PEXEL motif in sequences of the PfEMP1 protein family (red), exported proteins with a predicted signal peptide (blue), and exported proteins lacking a predicted signal peptide (gray). In B) the blue bars show the positions of the PEXEL motif after cleaving off the predicted signal peptide. Only sequences with a predicted signal peptidase cleavage site (*score* >0.5 according to SignalP [38], [39]) are included. Gray bars in B) represent the unchanged distribution of PEXEL in proteins lacking a predicted signal sequence. Note that all bars are displayed on top of each other.

A further hint towards a family-specific function of the N-terminal flanking region is that, according to our analysis, only 24% of the proteins with a PEXEL motif actually possess a standard signal sequence. It has been reported that PEXEL is preferably located 15–20 amino acids downstream of an N-terminal hydrophobic signal sequence [2], [3]. In Figure 4a, the PEXEL motif distribution in our set of 412 proteins is shown. We observe three groups of sequences with preferences around positions 20, 43, and 85. All PfEMP1 proteins lack a standard signal peptide, and the PEXEL location is near the protein N-terminus between residue positions 12 and 28. In contrast, in proteins containing a predicted signal peptide we find the PEXEL motif in a range of approximately 30 residues, between positions 37 and 63. We then artificially cleaved off the signal peptide in precursors with a predicted cleavage site and analyzed the resulting mature proteins: PEXEL motifs shift to positions 13–29, which is now comparable to the position of the PEXEL motif in PfEMP1 proteins lacking a signal peptide (Figure 4b).

The group of proteins with a PEXEL preference around position 85 does not contain a canonical signal peptide, but rather a recessed N-terminal hydrophobic segment, which has previously been shown to function as an ER targeting signal [2], [33], [39]. The proteins with a PEXEL preference around positions 35–50 are predicted to be exported as they, in addition to a PEXEL sequence, possess a hydrophobic N-terminal segment (Figure 4b, gray bars). Many of these proteins may actually contain an export signal which is not recognized by SignalP. As no standard algorithm predicts cleavage of these sequences and it is unclear whether these sequences are actually cleaved at all, no shift in the position of PEXEL is predicted in the analyses shown in Figure 4b.

These analyses support the hypothesis that, although different mechanisms may exist for initial entry of PEXEL containing proteins to the secretory pathway, mediated either by an N-terminal signal sequence, or another, as yet uncharacterized mechanism, certain positional constraints are exerted on the PEXEL motif, potentially related to the nature of the protein translocation machinery. As a consequence, recessed signal sequences−such as those present in glycophorin-binding protein 130 (GBP130) and the ring-infected erythrocyte surface antigen (RESA)−might be actually cleaved to bring the PEXEL motif into the correct positional preferences required for further transport.

On this note, the strong conservation of the initial arginine residue in the PEXEL motif is of interest. Arginine residues can often be found in protein targeting motifs such as the TAT (twin arginine translocation) signal peptide [41], and arginine based ER retention signals. It is possible that the arginine residue in the PEXEL motif associates the exported protein with the membrane of the parasitophorous vacuole prior to passage through the putative translocon. Such membrane binding properties have recently been shown for arginine residues in the TAT signal [42].

Summarizing, we found conserved hydrophobicity profiles rather than conserved residue patterns in the PEXEL-flanking regions. This hints toward potential recognition of the PEXEL motif and flanking regions by an interacting macromolecule and supports earlier experimental findings [12]. Any conserved property profile most likely is a result from gene duplication and other evolutionary events leading to the formation of different protein families. Bioinformatical analysis alone will not be able to undoubtedly determine whether these patterns are part of a PEXEL-related targeting signal or responsible for a completely different function. Still, our study provides a well-motivated basis for the necessary biochemical experiments. Although we may use the PEXEL motif to speculate about the nature of the *P.falciparum* “exportome”, we are only now beginning to understand the processes governed by this sequence, their biological importance, and how such processes are regulated, possibly by residues directly abutting the PEXEL sequence itself.
